# Ab Initio Electronic, Magnetic, and Optical Properties of Fe Phthalocyanine on Cr_2_O_3_(0001)

**DOI:** 10.3390/molecules29122889

**Published:** 2024-06-18

**Authors:** Marco Marino, Elena Molteni, Simona Achilli, Giovanni Onida, Guido Fratesi

**Affiliations:** ETSF and Physics Department “Aldo Pontremoli”, University of Milan, Via Celoria 16, 20133 Milan, Italy; elena.molteni@unimi.it (E.M.); simona.achilli@unimi.it (S.A.); giovanni.onida@unimi.it (G.O.)

**Keywords:** organic spintronics, spinterface, iron phthalocyanine, Cr_2_O_3_(0001) surface, DFT+U, optical spectroscopy

## Abstract

The organic molecules adsorbed on antiferromagnetic surfaces can produce interesting interface states, characterized by charge transfer mechanisms, hybridization of molecular-substrate orbitals, as well as magnetic couplings. Here, we apply an ab initio approach to study the adsorption of Fe phthalocyanine on stoichiometric Cr_2_O_3_(0001). The molecule binds via a bidentate configuration forming bonds between two opposite imide N atoms and two protruding Cr ones, making this preferred over the various possible adsorption structures. In addition to the local modifications at these sites, the electronic structure of the molecule is weakly influenced. The magnetic structure of the surface Cr atoms shows a moderate influence of molecule adsorption, not limited to the atoms in the close proximity of the molecule. Upon optical excitation at the onset, electron density moves toward the molecule, enhancing the ground state charge transfer. We investigate this movement of charge as a mechanism at the base of light-induced modifications of the magnetic structure at the interface.

## 1. Introduction

The use of molecular compounds in the field of spintronics [1,2] has been motivated by their low SOC and small carrier mobility, which are due to the charge hopping as the main transport mechanism [3]. From here, new phenomena have emerged, such as the negative magnetoresistance effect in organic spin valves [4,5] and the magnetic hardening in antiferromagnetic substrates induced by molecule adsorption [6]. The key aspect of the newly discovered phenomena of these organic–inorganic heterostructures [7] lies at the interface [8], where orbital hybridization occurs [9], which can modify the properties of the adjacent systems. This has led to the rise in spinterface science, addressing fundamental properties and applications of this kind of interface [10].

Antiferromagnetic substrates have appealing characteristics for the realization of spintronic devices (e.g., a minimal dissipative coupling to external magnetic stimuli) [11], and antiferromagnetic spinterfaces with, e.g., spin crossover molecules [12] can be designed. Transition metal oxides with a high Néel temperature may combine antiferromagnetism with insulating behavior, propagating spin excitations but blocking stray currents.

Different studies have already been carried out on the adsorption of organic molecules on oxides, such as In_2_O_3_ [13] and SrTiO_3_ [14], and transition metal oxides, such as TiO_*x*_, MoO_*x*_ [15], CoO [16], MnO [17], and NiO [18], focusing on structural deformations, charge transfer mechanisms, magnetic interactions, and the hybridization of the molecular and substrate orbitals. The insulating characteristic and the local magnetism of these substrates produce effects that are at variance with those observed in molecular adsorption on metallic surfaces [19,20,21], such as a typically smaller hybridization of the molecular orbitals with the substrate ones, allowing the molecule to preserve its characteristics to a larger extent. Among the transition metal oxides, Cr_2_O_3_, in its antiferromagnetic G-type phase [11], represents an interesting candidate as a substrate due to its characteristics being in between a charge transfer and a Mott–Hubbard insulator [22]. Among the large variety of molecular compounds, instead, organic molecules with metallic centers, such as metal phthalocyanines (Pcs) or metal porphyrins [23,24,25], can facilitate the magnetic coupling with the substrate [26,27], making them optimal candidates for building spinterface heterostructures. The possibility of modifying the metal center and the functional groups allows for a variety of chemical and physical phenomena [25]. For the formation of stable and ideally well-ordered molecular layers that are more directly accessible through surface science studies, it is also desirable to anchor the molecules at the surface, in addition to van der Waals forces, via direct chemical interaction or involving an appropriate chemical group that could act as an anchor. In this respect, it was previously proven that phthalocyanine molecules can bind in a selective way to a SiC surface that exposes adatoms with dangling bonds at a separation compatible with that of opposite imide nitrogens [28,29], which is also the distance between next-nearest Cr atoms of Cr_2_O_3_(0001).

Here, we study the adsorption of Fe phthalocyanine (FePc) on a Cr_2_O_3_(0001) substrate through ab initio methods based on Hubbard-corrected density functional theory (DFT), determining the mechanisms of adsorption and the magnetoelectrical properties of the interface. We identify a stable adsorption configuration where FePc bonds with external Cr through two imide N atoms, forming a bridge-like structure analogous to the one found on SiC surfaces. We further evaluate the optical spectra of the adsorbed system and analyzed it, thoroughly looking for the possibility of coupling between the light and the spin properties of the system.

## 2. Results

### 2.1. Adsorption Configurations

**Figure 1:**
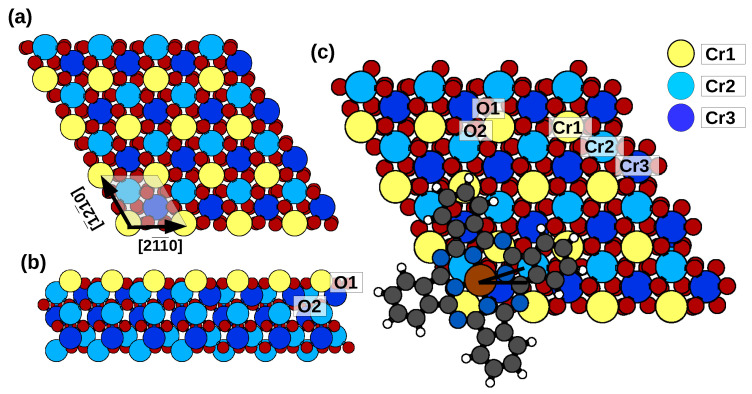
(**a**) Top view and (**b**) side view of the 4×4 surface supercell used for the calculations, with primitive cell of Cr_2_O_3_(0001) indicated in (**a**) (shaded). (**c**) Model of FePc/Cr_2_O_3_(0001), indicating the angle between the N-Fe-N axis and the surface $\left[2\bar{1}\bar{1}0\right]$ direction (Black lines centered on Fe atom). Color scheme as follows: yellow: topmost Cr atoms (named Cr1); light and dark blue: Cr atoms of the subsurface trilayer, according to their respective height (light higher Cr2, dark lower Cr3) in the bilayer 3O-2Cr-3O; red: O atoms. The magnetization of the Cr3 is parallel to that of the Cr1 atoms and opposite to that of the Cr2 atoms. Three of the O atoms are in the surface trilayer (O-up) and three in the subsurface trilayer (O-dn). Brown: Fe.

The Cr_2_O_3_(0001) surface can display different terminations; here, we model a stoichiometric one, terminated by Cr atoms (-O_3_-Cr), which appears to be the most stable one under a wide range of experimental conditions [30]. Interestingly, this termination exposes the Cr atoms of a specific magnetization [30,31,32,33]. We consider Cr_2_O_3_ in its antiferromagnetic phase (G type, see Figure 1), with a theoretical lattice parameter of 5.10 Å, which is slightly larger than the experimental value (3%) but in close agreement to that in a previous numerical study [30]. To study the adsorption of individual FePc molecules on Cr_2_O_3_(0001), we place one molecule on a 4×4 surface supercell, which corresponds to a distance of 20.42 Å between the metallic centers of nearby repeated molecules and of 5.48 Å between the facing H atoms. In this way, the molecules are reasonably diluted compared to those of packed phthalocyanines on Al(001), whose overlayer lattice parameter is 14.28 Å, as measured by low-energy electron diffraction [34].

**Figure 2:**
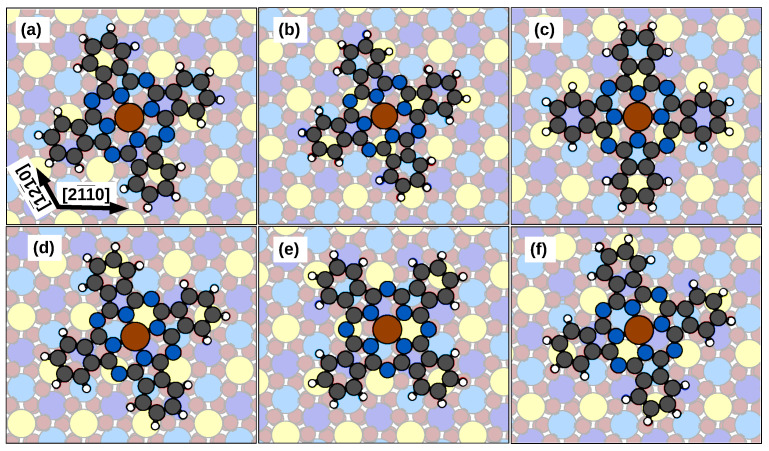
Optimized geometry for FePc/Cr_2_O_3_(0001) for the adsorption configurations considered: (**a**) on a Cr1 site at $22^{\circ }$, (**b**) on a Cr2 site at $22^{\circ }$, (**c**) on a Cr3 site at $0^{\circ }$, (**d**) on an O-up site at $22^{\circ }$, (**e**) on an O-dn site at $45^{\circ }$, and (**f**) on a B site at $15^{\circ }$. The color scheme is the same as in Figure 1.

Different adsorption configurations of planar molecules have been considered (see Figure 2), which are distinguished by their adsorption site, the azimuthal angle between one of the molecule axes and the substrate $\left[2\bar{1}\bar{1}0\right]$ direction, and the magnetic configuration, i.e., with the Fe spin being either ferromagnetic (FM) or antiferromagnetic (AF) with respect to the spin of the underlying Cr1 atom (see below). Concerning the adsorption sites, we start the geometry optimizations with the Fe atom placed above all the surface atoms that are visible from the top: a topmost Cr atom (Cr1), the two Cr atoms below the O_3_ layer (Cr2 and Cr3, from top to bottom), an O atom of the first layer (O1) or of the second one (O2), and the bridge site between two Cr1 atoms (B).

The resulting adsorption energies are reported in Table 1. It emerges that the lower energies configurations (e.g., (b,d–f) reported in Figure 2) are related to the possibility of the molecule bonding to the Cr1 atoms through the imide N atoms. Indeed, the adsorption energies of the O configurations are higher (by ≈0.6–0.7 eV) than those of the Cr configurations, with those molecular angles (O-up $45^{\circ }$ and O-dn 0°) favoring the bond (N-Cr1 ≈2.7 Å). The phenyl rings, in this regard, play a less significant role, as can be observed in the Cr1 configurations at angles of $0^{\circ }$ and $22^{\circ }$, where a smaller increase in the adsorption energy can be observed (≈0.4 eV).

The significance of the Cr1–N atom bonding is underlined by additional findings: by the Cr3 configurations at $22^{\circ }$ and $45^{\circ }$ where the optimized structure is equivalent to the bridge at $15^{\circ }$ and by the increase (0.7 eV) in the adsorption energy in the bridge configuration as the molecule rotates from $75^{\circ }$ to $15^{\circ }$, which is the configuration with the most significant overlap between the imide N and the Cr1 atoms.

On the other hand, while the energetics depend on the bonding between the Cr1 and N atoms, the magnetic behavior of the molecule seems to depend on the position of the Fe atom with respect to the surface. The proximity of the Fe atom to Cr1 or Cr3 apparently favors a ferromagnetic coupling, while the other configurations seem to favor an antiferromagnetic superexchange coupling through the O atoms with the atoms below. In particular, in the bridge configuration at $15^{\circ }$ the molecule, with an imide N–Cr1 bonding of 2.47 Å, shifts partially toward the O-dn site, hence approaching a Cr1 atom (that on the bottom left of the Fe atom). This shift enables a lower Fe atom height of 2.62 Å above the Cr1 layer and the consequent bonding with the nearby Cr1 (on the contrary, the opposite shift toward the O-up atom would be 0.28 eV higher in energy). This proximity involves a ferromagnetic ordering of the Fe magnetic moment with respect to the Cr1 magnetization.

### 2.2. Electronic Properties

**Figure 3:**
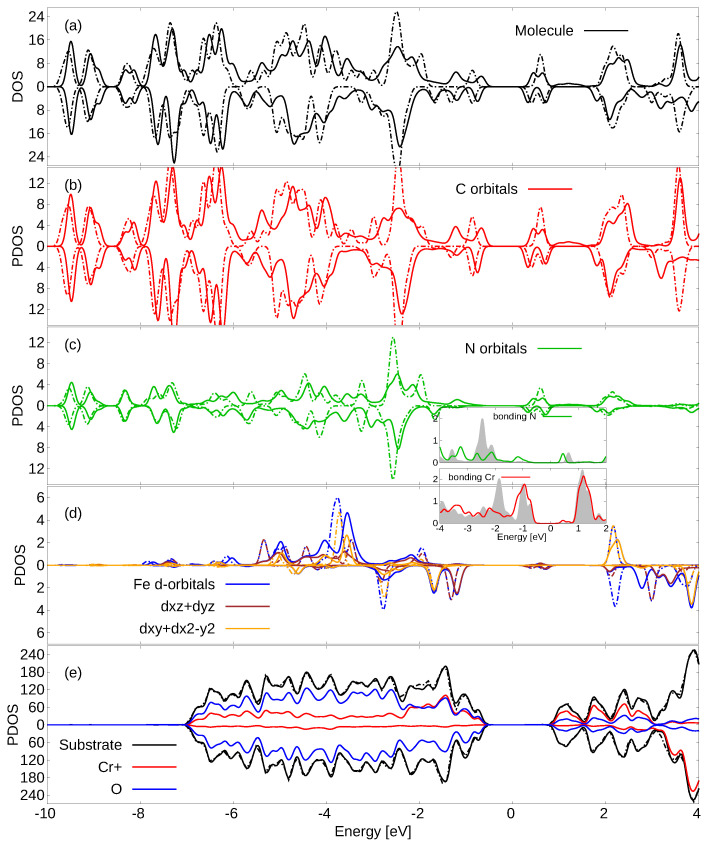
Electronic PDOS of FePc/Cr_2_O_3_(0001) summed over (**a**) all molecule atoms; (**b**) C; (**c**) N; (**d**) Fe; (**e**) substrate atoms. Substrate color codes: black: all substrate atoms; red: all Cr atoms with “up” magnetic moment; blue: all O atoms. Solid/dash-dotted lines indicate adsorbed and gas phase molecules, respectively. Spin-down components are shown as negative values. All values in states/eV/cell. The position of the Fermi energy in the gap is arbitrary. In the inset, we report the spin-up contributions from the bonding imide N and Cr1 (lines) atoms over the other imide N and Cr1 atoms (gray area).

Here, we focus on the most stable adsorption configuration (bridge FM at $15^{\circ }$). We discuss its electronic properties by looking at the projected density of states (PDOS) reported in Figure 3.

The LUMO of the molecule, inside the valence–conduction gap of the pristine substrate, appears split in the spin-up channel and at lower energy than its counterpart in the free molecule (taking deep molecular orbitals as a reference). Although the HOMO of FePc is slightly lower in energy than the valence band maximum, there is overall a reduction in the valence-conduction gap of the adsorbed system. The spin-up channel valence-conduction gap reduces from 1.67 to 1.07 eV, while the spin-down channel one lowers from 1.66 to 1.09 eV.

In the adsorbed system, we refer to unoccupied Kohn–Sham states as “lu + *n*” and to occupied ones as “ho − *n*” (where lu and ho are the first unoccupied and last occupied states, which in the case of the molecular system correspond to the usual LUMO and HOMO). Here, we underline that in the spin-up channel of the ho, and the lu and lu + 1 (+0.2 eV above the lu) have molecular characteristics (50% and ∼81%, respectively), while in the spin-down channel, only the lu and the lu + 1 (at 0.38 eV above lu) have molecular characteristics (∼94%). The other states in proximity of the band gap, the lu +n and the ho −n, instead have substrate characteristics (>90%). Additionally, the ho −9 in the spin-down channel (−0.04 eV below the HOMO) has molecular characteristics (87%).

Further away from the band gap, there are additional orbitals with molecular characteristics in the spin-up channel at −0.52 eV below the ho (15%) and at +1.56 eV above the lu (50% of which 26% of Fe nature), and in the spin-down channel at −0.48 eV below the ho (65%, of which 40% of Fe nature) and at 1.47 eV above the lu (66%). Thus, out of the molecular orbitals mentioned above, the ones in proximity to the band gap mostly originate from the C and secondarily from the N atoms of the organic macrocycle C, whereas they show Fe contributions only further away from the Fermi energy.

**Figure 4:**
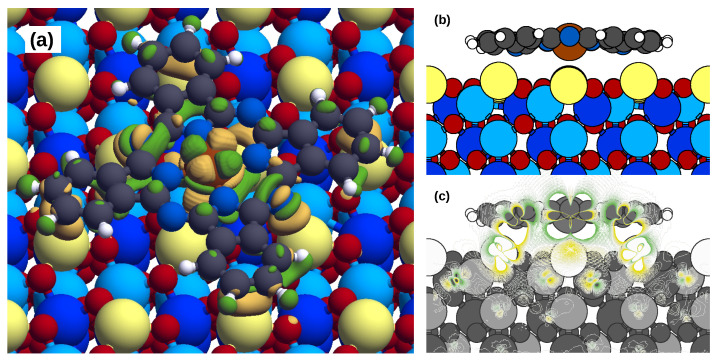
On the left, (**a**) top view; on the right, (**b**,**c**) side-view of the charge density variation in the FePc/Cr_2_O_3_(0001) minimum energy adsorption configuration (isovalues in the range of 0.001 Å × $10^{-3}$). Note the bondings between the imide N and the Cr1 and between the phenyl rings and the Cr1. Color scheme the same as in Figure 1.

The d orbitals of the Fe atom are shifted and broadened; however, their energy position is partially preserved. In our previous study of FePc/NiO(001) [18], due to the major relevance of the Fe–substrate bonding, the perturbation of the orbitals along the direction normal to the surface ($d_{z^{2}}$, $d_{xz}$, and $d_{yz}$) was more significant. Overall, the changes in the PDOS of the molecule and of the substrate are moderate. Noticeable exceptions are those visible at energies below the ho in the N and C orbitals, which are involved in the bonding with the Cr1 atoms. To see this, we report their PDOS as an inset in Figure 3, focusing on the spin-up component, as the spin-down Cr DOS is much lower at those energies. We can see modification of the DOS and energetic correspondence of some peaks; see, for example, at about −3, −2 and −1 eV, which are not present for the other imide N and Cr1 atoms not forming the bonds. The bonding between these two atoms is especially evidenced by the real-space charge density, which is shown in Figure 4.

### 2.3. Magnetic Properties

**Figure 5:**
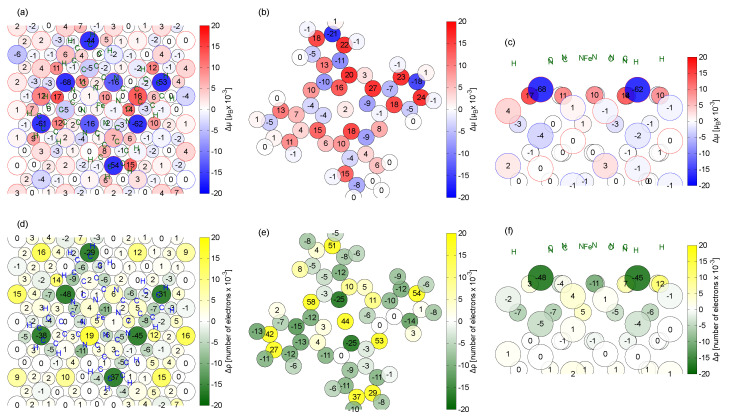
Variations in the atomic magnetic moments of FePc/Cr_2_O_3_(0001), seen from the top, with respect to the isolated systems, on (**a**) the surface and on the molecule (**b**). Corresponding variations in the electronic density (**d**,**e**). (Positive = increase in electron population.) Side view of the variations: (**c**) magnetic moments and (**f**) charge density. The positions of the molecule atoms are marked by green/blue letters in panels (**a**,**c**,**d**,**f**). Notice that few values in the plot exceed the maximum ones on the scale bar.

The adsorption of the molecule modifies the magnetic structure of the system. In Figure 5, we report the modifications of the local magnetic moments of the substrate (panel a and c) and of the molecule (panel b) following adsorption, as computed via Löwdin population analysis.

We find that the molecule increases its magnetic moment by 0.19 $\mu_{B}$, which is balanced by an opposite variation in the substrate. Looking to the substrate, the variations are mainly localized in the top surface layer, with small contributions that also extend to the subsurface layers (see the side view in Figure 5c) but decay rapidly. These small variations underline that the magnetic interaction between the molecule and the substrate is not propagating through the substrate, as instead happens when the same molecule is adsorbed on the NiO(001), mediated by superexchange mechanisms [18]. Note that the most affected Cr is the Cr1 bonding to the imide N atoms ($\Delta \mu \approx -60\times 10^{-3}$ $\mu_{B}$) or under the phenyl rings ($\Delta \mu \approx -20\times 10^{-3}$ to $-60\times 10^{-3}$ $\mu_{B}$). Additional analysis of the electron density alteration upon adsorption (namely, the bond charge depicted in Figure 4, here projected onto atoms) is shown in Figure 5d–f. Partial matching of the patterns with those of panels a–c demonstrates that the magnetic variations here discussed are strictly related to the electron density variations. The atoms of the substrate participating in the C-Cr1 and N-Cr1 bonds lose electrons (≃$38\times 10^{-3}$ $e^{-}$), while those of the molecule acquire electrons (≃$40\times 10^{-3}$ $e^{-}$). This movement of electrons is related to the spin-up channel, thus implying a reduction in the local magnetic moments in the top-most substrate atoms (Cr1) (≃$40\times 10^{-3}\;\mu_{B}$) (note that the clean surface magnetic moments for Cr1 and Cr3 are 3.14 $\mu_{B}$ and for Cr2 is −3.15 $\mu_{B}$). The molecule acquires 0.190 electrons overall, which are not evenly distributed; the external imide N atoms and the C atoms acquire, respectively, 0.105 and 0.143 electrons; the internal pyrrolic N atoms and the H atoms lose, respectively, 0.053 and 0.049 electrons. No significant variation in the Fe magnetic moment (free molecule magnetic moment 2.17 $\mu_{B}$) is observed despite its alteration in electron density because this charge transfer involves mostly states with *s* symmetry.

### 2.4. Optical Properties

**Figure 6:**
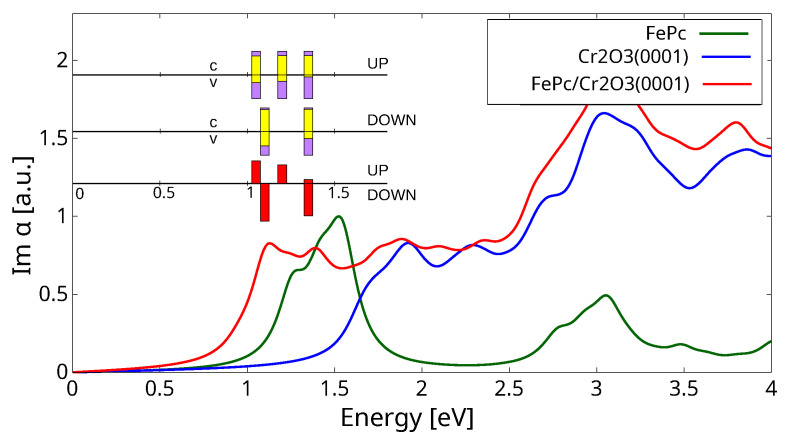
Optical absorption spectra (independent particle): polarizability of Cr_2_O_3_(0001) (blue), FePc/Cr_2_O_3_(0001) (red), and an FePc molecular layer such as in FePc/Cr_2_O_3_(0001) (green). The arbitrary units are chosen by normalizing the molecular peak at ≃1.5 eV (green line) to 1. The transitions are broadened by 0.01 eV. Bottom inset: the intensities (red) of the main transitions contributing to the FePc/Cr_2_O_3_(0001) spectrum in the spin-up (top) and spin-down (bottom) channels, normalized with respect to the largest one at ≃1.1 eV. Only values greater than 10% are shown. Top inset: the percentage of projection of the respective valence(bottom)/conduction(top) level on the molecule (yellow), characterizing the main transitions.

We now discuss the optical properties of FePc/Cr_2_O_3_(0001) in the minimum energy adsorption configuration, reporting 2D polarizability in Figure 6. There, we also report the corresponding spectra of FePc (in a molecular layer having the same unit cell and coordinates to facilitate comparisons) and of the clean substrate. We remark that the intensities of the three spectra were scaled by the same factor, chosen so as to normalize the transition in the free molecule at ≃1.5 eV to unit intensity. We can observe transitions in the free molecule at energies lower than the onset of the substrate. Interestingly, the spectral onset in the full system occurs further on at lower energy; this is due to the hybridization of the molecular and substrate orbitals and the consequent reductioninof the band gap, as is clear in the PDOS (Figure 3).

This behavior is now analyzed by performing a projection of the valence–conduction states, so as to single out the corresponding molecular contributions. Namely, for each transition, we sum the squared projections of the valence states $\psi_{\nu }$ involved in the atomic orbitals $\phi_{nlm}$ of atom *J*, $w_{\nu }^{\left(J\right)}=\sum_{nlm}{\left|\langle \phi_{nlm}^{\left(J\right)}|\psi_{\nu }\rangle \right|}^{2}$, taking *J* in the molecule, and we weight this by the transition intensity $I_{t}$. Since many transitions contribute in similar energy ranges, we group transitions *t* into bin *b* with a binning of 0.05 eV and obtain the molecular projection within a given bin as:

(1) $$P_{b}^{\left(v\right)}=\left\{\sum_{t\in b}I_{t}\left[\sum_{J\in \mathrm{FePc}}w_{v(t)}^{\left(J\right)}\right]\right\}/\left\{\sum_{t\in b}I_{t}\right\}$$

and similarly for conduction states $\psi_{c}$. In the lower inset in Figure 6, we report in red the relative intensity of the transitions in the the two spin-channels, separately, normalized with respect to the maximal one at ≃1.1 eV (bins with relative intensity <10% are omitted). In the upper inset, we show the molecular contributions to the valence and conduction states, $P_{b}^{\left(v\right)}$ and $P_{b}^{\left(c\right)}$, respectively, below and above the straight line for each channel in yellow.

The first part of the spectrum (<1.50 eV) has a resemblance to the energy-shifted free-molecule one, in addition to a partial breaking of the degeneracy in the spin-up channel, where a nearly degenerate peak at energies 1.46–1.47 eV [18] shifts to a double peak at 1.09 and 1.24 eV, as induced by the interaction with spin-up polarized Cr1 atoms. Despite this similarity, we notice that the valence levels of the transitions have a significant contribution to the substrate (40–70%). Compared to this finding, in our previous study of FePc/NiO(001) [18], the substrate contribution to the valence levels of the transitions was less significant, not exceeding 40%. Conversely, the conduction levels involved in the transitions have a dominant molecular characteristic (≥80%). This implies that, upon excitation, electron density is displaced from the substrate toward the molecule. The difference in the spin-up and spin-down channels is attributed to the spin polarization of the surface atoms under the molecule, as the topmost Cr atoms are all of the Cr1 type (spin-up polarized), see Figure 2. So, despite of the overall spin- symmetry of the substrate DOS in Figure 3, the spectrum shows a broken spin symmetry.

As a further analysis, we approximately evaluate the average height with respect to the surface of the Kohn–Sham states contributing the transitions in a given bin as

(2) $$z_{b}^{\left(v\right)}=\left\{\sum_{t\in b}I_{t}\left[\sum_{J}z^{\left(J\right)}w_{v(t)}^{\left(J\right)}\right]\right\}/\left\{\sum_{t\in b}I_{t}\right\}$$

for valence states and similarly for conduction ones, $z_{b}^{\left(c\right)}$. That is, we estimate the average *z* coordinate of the electrons before and after the optical excitation, taking z=0, the height of Cr1 atoms, as a reference. Considering both main peaks in the spin-up channel, the weighted height of the valence levels amounts to $z^{\left(v\right)}=-0.7\pm 0.1$ Å, that is, between the Cr1 and top bilayer Cr2-Cr3 atoms, while that of the conduction levels is $z^{\left(v\right)}=1.4$ Å, i.e., 1.3 Å below the molecule. Considering instead the two main peaks in the spin-down channel, we have $z^{\left(v\right)}=0.1$ Å and $z^{\left(v\right)}=-1.5$ Å, i.e., close to the top-surface Cr1 atoms and at the top-bilayer Cr2-Cr3 atoms, and $z^{\left(c\right)}=2.1$ Å for both, so 0.6 Å under the molecule. This suggests a movement of charge from the top layers of the substrate toward the molecule.

Looking within the molecular contributions, the valence states do not have appreciable contributions from the Fe atom, while the conduction states have moderate contributions (a few %), which differs in the two spin channels: ≃1.4% in the spin-up one and 3–5% in the spin-down one. Thus, not only a partial localization of charge on the Fe atom can be inferred, but also, taking into account the larger intensity of the peaks in the spin-down channel, a plausible small decrease in the Fe magnetic moment.

## 3. Computational Methods

We evaluate the ground state and its properties using DFT in a plane-waves basis set, as implemented in the Quantum ESPRESSO distribution (6.8) [35,36]. Hubbard corrections are included in their Dudarev formulation (a single parameter is given $U_{eff}=U-J$) to describe on-site correlation effects of the transition metal atoms. This approach introduces some level of arbitrariness about the choice of the additional Hubbard *U* parameter, which inevitably influences the results. However, in our specific case, the adsorption energy is primarily dominated by the formation of bonds between the imide N atoms and surface Cr1, with a large energy difference with respect to the other cases. So, we are confident this result is robust with respect to the choice of *U*. The values considered, $U_{Cr}=4.0$ eV and $U_{Fe}=5.0$ eV, were chosen following the literature so as to optimize the electronic properties (density of states) of the localized orbitals for the isolated systems [18,30]; with these values, the Cr_2_O_3_ characteristic, intermediate between a charge transfer insulator and a Mott–Hubbard insulator, is well reproduced, as well as the magnetism of the Fe atom of the molecule. We include the van der Waals interactions between the molecule and the substrate by using the vdW-DF-C09 exchange and correlation (xc) functional [37,38]. Vanderbilt ultrasoft pseudopotentials (GBRVs) [39] with semicore corrections are used, selecting a plane wave cutoff of 45 Ry for the wave functions and of 270 Ry for the charge density. Given the large supercell with lattice vectors of 2.04 nm, the surface Brillouin zone is sampled by the Γ point only. The substrate is modeled with a slab that includes 4 Cr_2_O_3_ layers. Equilibrium geometries are obtained through a structural optimization of the molecule and of the top three layers of the substrate.

The optical spectra of the system are associated with the 2D polarizability, obtained through the imaginary part of the dielectric function. This is evaluated at the independent particle (IP) level through the matrix elements of the dipole operator between the Kohn–Sham states. These calculations were performed using the Yambo code (5.1) [40], a plane-wave code interfaced with Quantum ESPRESSO. Since Yambo is not compatible with ultrasoft pseudopotentials or with VdW-DF-C09 exchange correlation functionals, additional calculations at fixed atomic coordinates were performed with norm-conserving pseudopotentials (Pseudo-Dojo [41]) and the Perdew–Burke–Ernzerhof (PBE) [42] exchange correlation, including as well the Hubbard corrections as specified above. We verified the consistency of the electronic and magnetic structure with that obtained before. In the dielectric function evaluation, we considered the Hubbard correction to the Kohn–Sham eigensystem as described above, but we computed approximate matrix elements by neglecting the contribution by the Hubbard Hamiltonian while evaluating the commutator [r,H]. We verified in a previous work that this approach yields qualitatively analogous results with respect to the full calculation, at a lower computational cost [18].

## 4. Conclusions

We studied the adsorption of Fe phthalocyanine on Cr_2_O_3_(0001) in its stoichiometric termination and antiferromagnetic phase. Among the adsorption configurations, the adsorption is stabilized by a specific interaction between the two opposite imide N atoms of the molecule and the topmost Cr atoms of the surface, additionally via interaction with the phenyl rings. Due to the small differences in energy between the configurations still bridging the Cr atoms, the detailed centering of the molecule can vary between the O of the topmost CrO_3_Cr trilayer and the O of the second one. The electronic structure mainly shows changes at the bonding atoms, weakly affecting the other ones. The molecule influences the magnetic moment of the surface atoms, especially those that are directly bound to the molecular N or phenyl rings. Overall, a moderate charge transfer from the substrate toward the molecule is observed. From the perspective of the optical spectra, the first peaks in the combined system can be found at energies below the absorption thresholds of both the free molecule and the clean substrate and are ascribed to transitions involving a significant contribution of the substrate orbitals toward the molecular ones. This extra density sums up to that transferred upon adsorption, with alteration of the interface magnetism: while it implies a reduction in the spin-majority electrons of the surface Cr atoms, it leads to a small decrease in the magnetic moment of the Fe atom. This study further shows the range of the physics and chemistry of tetrapyrrole molecules at magnetic surfaces, crucially depending on both the substrate and adsorbate for their specific properties. 

## Figures and Tables

**Table 1:** Adsorption energy for FePc/Cr_2_O_3_(0001) as a function of adsorption site and optimized azimuthal angle for FM and AF orientation of the Fe spin with respect to underlying Cr1 atoms. Values in eV.

Site	Angle	$E_{\mathrm{ads}}^{\mathrm{FM}}$	$E_{\mathrm{ads}}^{\mathrm{AF}}$	$E_{\mathrm{ads}}^{\mathrm{AF}}-E_{\mathrm{ads}}^{\mathrm{FM}}\;\left[\mathrm{eV}\right]\;$
Cr1 (up)	$0^{\circ }$	−3.793	−3.710	0.082
	$22^{\circ }$	−3.453	−3.860	−0.407
Cr2 (up)	$0^{\circ }$	−3.436	−3.519	−0.083
	$22^{\circ }$	−3.533	−3.541	−0.009
Cr3 (down)	$0^{\circ }$	−3.493	−3.438	0.055
O-up	$0^{\circ }$	−4.100	−4.165	−0.065
	$22^{\circ }$	−4.202	−4.203	−0.001
	$45^{\circ }$	−3.558	−3.575	−0.017
O-dn	$0^{\circ }$	−3.444	−3.454	−0.010
	$45^{\circ }$	−4.233	−4.234	0.000
B	$15^{\circ }$	−4.316	−4.252	0.065
	$24^{\circ }$	−4.169	−4.234	−0.065
	$45^{\circ }$	−3.444	−3.460	−0.016
	$52^{\circ }$	−3.494	−3.511	−0.016
	$75^{\circ }$	−3.410	−3.394	0.016

## Data Availability

Dataset available on request from the authors.
